# Biochemical and Toxinological Characterization of Venom from *Macrorhynchia philippina* (Cnidaria, Hydrozoa)

**DOI:** 10.1155/2022/8170252

**Published:** 2022-05-17

**Authors:** Karine Cristie Quaglio Banagouro, Jefferson Viana, Leonardo Pereira de Lima, Guilherme Rabelo Coelho, Thalita Rocha, Raquel Girardello, Karolayne Larissa Russi, Marcelo V. Kitahara, Juliana Mozer Sciani

**Affiliations:** ^1^Unidade Integrada de Farmacologia e Gastroenterologia-UNIFAG, Universidade São Francisco, Bragança Paulista 12916-900, Brazil; ^2^Programa de Pós-graduação Stricto Sensu em Ciências da Saúde, Universidade São Francisco, Bragança Paulista 12916-900, Brazil; ^3^Laboratório Multidisciplinar de Pesquisa, Universidade São Francisco, Bragança Paulista 12916-900, Brazil; ^4^Laboratório de Bioquímica e Biofísica, Instituto Butantan, 05503-900, Brazil; ^5^Programa de Mestrado em Biomateriais e Medicina Regenerativa, Pontifícia Universidade Católica de São Paulo, Sorocaba 18030-070, Brazil; ^6^Laboratório de Microbiologia Molecular e Clínica, Universidade São Francisco, Bragança Paulista 12916-900, Brazil; ^7^Centro de Biologia Marinha, Universidade de São Paulo, São Sebastião 11600-000, Brazil; ^8^Departamento de Ciências do Mar, Universidade Federal de São Paulo, Santos, 11015-020, 22, Brazil; ^9^Department of Invertebrate Zoology, Smithsonian Institution, Washington, DC, 20560-0163, USA

## Abstract

*Macrorhynchia philippina* is a colonial benthic hydroid from the Class Hydrozoa (Phylum Cnidaria) distributed in the tropical and subtropical marine waters from Atlantic Ocean, Indo-Pacific, and Mozambique. Its colonies somewhat resemble plants, causing confusion in the bathers who accidentally touch the animal. Acute burning/local pain, edema, erythema, and pruritus were symptoms already described, but its venom composition is unknown, as well as the participation of toxins for the symptom's development. Thus, herein, we show the biochemical composition and toxic effects of *M. philippina* venom. Colonies were collected and processed for histological analysis; alternatively, they were immersed into methanol containing 0.1% acetic acid for venom attainment, which was analyzed by mass spectrometry and submitted to edema and nociception evaluation in mice, hemolysis and antimicrobial assays *in vitro*. Before the molecule's extraction, it was possible to see the inoculation structures (hydrocladiums and hydrotheca) containing venom, which was released after the immersion of the animal in the solvents. The venom was composed mainly by low molecular mass compounds, able to cause significant reduction of the paw withdrawal latency from the hot plate test, 30 minutes after the injection. Moreover, significant edema was observed 10 and 30 minutes after the injection, indicating the activity of at least two inflammatory mediators. The venom caused no hemolytic activity but reduced the growth of *A. baumannii* and *K. pneumoniae* strains. This study is the first biochemical description of *M. philippina* venom, with molecules that cause fast inflammatory and painful effects, characteristic of the envenomation.

## 1. Introduction

The Phylum Cnidaria is a highly diverse group that includes the Class Anthozoa, Cubozoa, Hydrozoa, Myxozoa, Scyphozoa, and Staurozoa. All of them have venomous representatives, some with potent toxins for humans, injected in the victim through an inoculation apparatus, named nematocysts [1].

Belonging to the Class Hydrozoa, the genus *Macrorhynchia* comprises 26 extant colonial benthic hydroids distributed in tropical and subtropical marine waters [2]. Overall, their colonies somewhat resemble plants, causing confusion in the bathers who accidentally touch the animal.

The white-stinging-hydroid *Macrorhynchia philippina* has a circumtropical distribution, found in the Atlantic Ocean waters, Indo-Pacific area (Mediterranean and Red Sea), and Mozambique. It is commonly found on rocky shores along the Brazilian coast [2].

In general, accidents caused by *M. philippina* are characterized by acute burning/local pain, ranging from mild to severe, and could be accompanied by edema, erythema, and pruritus, but without systemic effects [3]. Although not lethal, its venom and the envenomation profile make this species harmful to divers and bathers [4, 5]. However, there are no studies describing its venom composition.

Similarly, venoms from other class, such as Cubozoa and Scyphozoa, commonly known as jellyfishes, frequently cause human envenomation. The most studied venomous hydrozoans are *Physalia physalis* and *Olindias sambaquiensis*, commonly found in Brazil [6, 7]. Furthermore, *Hydra* sp. and hydrocorals of the genus *Millepora* also contribute to envenomation cases [8].

Symptoms from Cubozoa and Scyphozoa are similar to those from *M. philippina*, and it is caused by the action of protein toxins, such as peptidases, neurotoxins, phospholipases, and ion-channel toxins [9]. Moreover, cytolysins were also identified from *Olindias sambaquiensis* [10]. Regarding enzymatic activity, metallopeptidases, serine peptidases, and phospholipases A_2_ were identified, being the phospholipase activity comparable to that from snakes [11].

Phospholipase A_2_ (PLA_2_) from *Millepora* was shown to have hemolytic activity, with proteins that contain hemolysins with or without PLA_2_ activity [12], such as Milleporin-1 [13] and MCTx-1 [14], respectively. Beside those, pore-forming toxins and neurotoxins are also found in Cnidaria venoms [15]. Low molecular mass compounds are also present in the Cnidaria venom, such as serotonin [16].

Although the symptoms of *M. philippina* envenomation have already been reported and it is typical of cnidarians, its venom composition is poorly known. Thus, herein, we investigated the venom of *M. philippina*, to characterize it biochemically and pathophysiologically.

## 2. Material and Methods

### 2.1. Sampling

**Figure 1:**
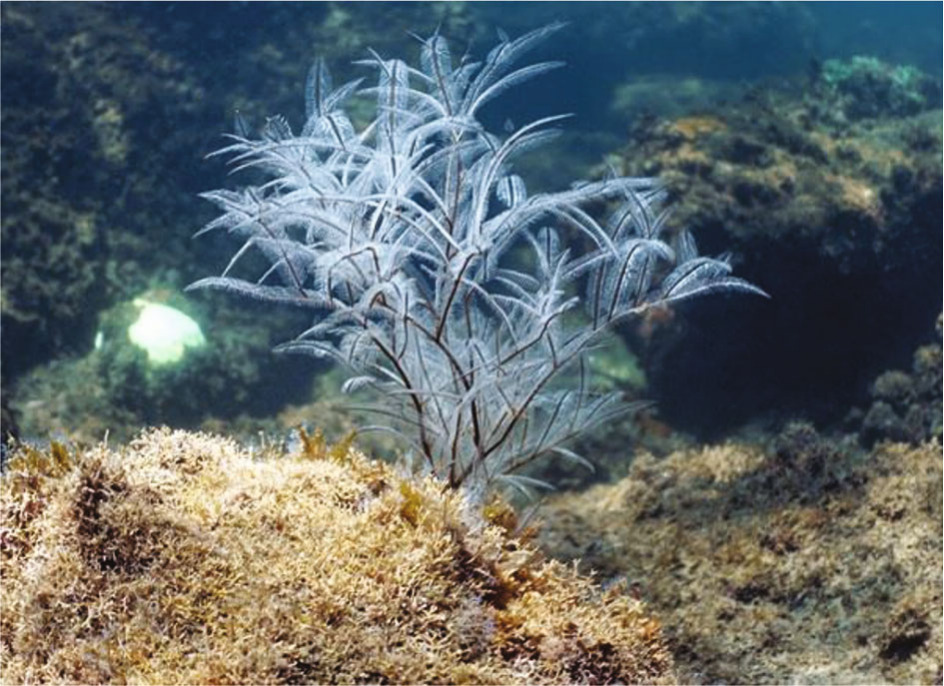
*Macrorhynchia philippina* (photo by Marcelo V. Kitahara).

*Macrorhynchia philippina* (Figure 1) specimens were collected by SCUBA diving at Ponta do Jarobá, São Sebastião, São Paulo, Brazil (23° 49′ 44^″^ S; 45° 25′ 23^″^ W) under the ICMBio license # 68917-1. Upon sampling, colonies were washed with filtered sea water and other animals and plants were manually removed.

### 2.2. Animal's Morphology

After cleaning, colonies were immediately fixed with Karnovsky's fixative solution (glutaraldehyde 3%, paraformaldehyde 3% in 0.1 M phosphate buffer, pH 7.4) for 48 hours. After fixation, the animals were examined using a light microscope and, in parallel, some colonies fragments were dehydrated in crescent ethanol series (70, 95, and 100%) and submitted to a xylene clarification process using an automatic tissue processor (Lupetec PT 09 TS) and embedded in paraffin (Lupetec DP 2010). Transversal or longitudinal 2 *μ*m sections were obtained using the microtome Lupetec MRPO3. Histological slides were deparaffinized in xylene, hydrated in decreasing concentrations of ethanol (100%, 95%, 80%, and 70%), hydrated in distilled water, and stained with hematoxylin-eosin (HE) for 5 and 3 minutes, respectively, and mounted with a slide and synthetic Canada balsam for general morphological observation under the light microscope (Nikon SE).

### 2.3. Venom Extraction

Fresh specimens of *M. philippina* were immersed in methanol containing 0.1% acetic acid for 24 hours at room temperature. Resulting extract was centrifuged at 10,000 × g for 10 minutes, and the supernatant was submitted to a rotary evaporator for solvent removal. The remaining content was then dissolved in the appropriate solution for subsequent analysis, described in the correspondent sections.

### 2.4. Biochemical Characterization

In order to verify the presence of proteins, *M. philippina* venom (50 *μ*g/mL) was submitted to an electrophoresis in a 12% polyacrylamide gel containing SDS (SDS-PAGE) performed under reducing conditions. Gel was later stained by silver.

Alternatively, the *M. philippina* venom (50 *μ*g/mL diluted in 100 mM Tris-HCl, pH 8.5) was added to 8 M urea or 15 minutes at 80°C, followed by the addition of dithiothreitol 100 mM for 30 minutes at 60°C and 300 mM iodoacetamide for 30 minutes at room temperature, protected from light. The solution was diluted with 100 mM Tris-HCl pH 8.5 to reach urea concentration to 2 mM and digested with 10 ng/*μ*L of trypsin (Proteomics Grade, from porcine pancreas, Sigma-Aldrich) overnight, at 37°C. The reaction was stopped by the addition of 5% formic acid, and the material was lyophilized to be analyzed in the mass spectrometry.

Aliquots were loaded in a C18 column (Phenomenex C18, 3 *μ*m, 100 Å, 1.0 mm × 50 mm) coupled to a reversed-phase ultraperformance liquid chromatography (Acquity, Waters Co., USA). The elution was performed in a two-solvent system: (A) formic acid (FA)/H2O (1 : 1000) and (B) FA/acetonitrile/H2O (1 : 900 : 100), at a constant flow rate of 0.2 mL/min with a 1–40% gradient of solvent B over 40 min. The column eluates were monitored automatically by mass spectrometry (Q-ToF Xevo GS, Waters Co.), in positive ionization mode, in a range of 200 to 2000 *m*/*z* and FWHM 40000 resolution at 500 *m*/*z*. For MS/MS analysis, argon collision energy was applied, and ions were monitored in a range of 100 to 1500 *m*/*z*.

The raw data was analyzed by PEAKS®7.0 software (Bioinformatics Solution Inc., Canada) for protein identification using the following parameters: MS and MS/MS error mass 10 ppm and 0.01 Da; methionine oxidation and carbamidomethylation as variable and fixed modification, respectively; trypsin as cleavage enzyme; 3 maximum missed cleavages and 3 maximum variable PTMs per peptide; and the false discovery rate ≤ 0.5%. All data were analyzed in a Cnidaria Database, built by retrieving all UniProt entries associated with this term.

In parallel, the venom was loaded into a C18 column (ACE C18, 5 *μ*m, 100 Å, 2.0 mm × 50 mm) coupled to a reversed-phase ultraperformance liquid chromatography (Acquity, Waters co., USA) to verify the complexity of the sample and the molecular mass distribution.

The elution was performed in a two-solvent system: (A) formic acid (FA)/H_2_O (1 : 1000) and (B) FA/acetonitrile/H_2_O (1 : 900 : 100), at a constant flow rate of 0.2 mL/min with a 0–100% gradient of solvent B over 40 min, after a 5 min isocratic elution with 0% B. The column eluates were monitored automatically by mass spectrometry (Q-ToF Xevo GS, Waters Co.), in a positive ionization mode, in a range of 200 to 2000 *m*/*z* and FWHM 40000 resolution at 500 *m*/*z*. For MS/MS analysis, argon collision energy was applied, and ions were monitored in a range of 100 to 1500 *m*/*z*.

Equipment control and data acquisition were conducted using the MassLynx 4.2. Raw files were manually verified and processed using PEAKS®7.0 (Bioinformatics200 Solution Inc., Canada) for peptide de novo sequence and Progenesis QI Software (Waters Co., USA) for small molecules, which were identified by exact molecular mass, *m*/*z*, and spectra similarity using the GNPS and Vaniya/Fiehn Natural Products databases. For both, 10 ppm was used as precursor and fragment tolerance. Moreover, ions previously described for *M. philippina* were manually searched in the peak list, in order to find known molecules in our extract.

### 2.5. Biological Activities

#### 2.5.1. Animals

Male Swiss mice (20-25 g) used throughout the study were treated under ethical conditions, following the current law from Conselho Nacional de Controle de Experimentação Animal (CONCEA) determinations, and maintained at the animal housing facilities of the Universidade São Francisco, approved by the Institutional Animal Care Committee of USF (CEUA, protocol number 007.11.2019).

Animals were placed into microisolators, in a room set at a 12/12 h light/dark cycle, with a 70% air humidity, and a constant temperature of 22°C. All animals had free access to potable water and food.

#### 2.5.2. Nociception Assay

Mice (*n* = 5) were injected by intraplantar route with *M. philippina* venom (10 *μ*g/paw, diluted in 30 *μ*L of sterile saline 0.9%) and evaluated by thermal stimuli to verify possible nociceptive activity of the secretion.

Before and after the injection (5, 15, 30, and 45 minutes), mice were placed in a hot plate set at a constant temperature of 50 ± 5°C. The response time of licking of a hind paw was recorded for each time, and the alteration of latency time with the *M. philippina* venom was compared to that measured previously.

#### 2.5.3. Paw Edema Assay

The *M. philippina* venom (10 *μ*g/paw, diluted in 30 *μ*L of sterile saline 0.9%) was injected in mice by intraplantar route (*n* = 6). The contralateral paw received the same volume of sterile saline 0.9% (control paw). Paw thickness was measured by a digital caliper at 0, 5, 10, 15, 20, 30, and 45 minutes after injection to verify possible edema.

Results were expressed as the difference (%) of thickness between *M. philippina*-injected paw and control paws.

#### 2.5.4. Hemolytic Activity

The hemolytic activity from *M. philippina* venom was tested in human erythrocytes obtained from four healthy volunteers (protocol approved by the Ethics Committee from Universidade São Francisco, CAAE 25441719.0.0000.5514). The blood, collected in EDTA tubes, was centrifuged at 1,000 × g for 10 min at room temperature. Red blood cells (RBC) were obtained, washed with phosphate-saline buffer (PBS, 50 mM, pH 7.3), and diluted to achieve a 4% suspension. The *M. philippina* venom (10 and 20 *μ*g, diluted in PBS) was incubated to 20 *μ*L of 4% RBC and 50 *μ*L PBS for 60 min at 37°C. After incubation, the resulting mixture was centrifuged at 4,000 × g for 5 min at room temperature and the supernatant has its absorbance (A) read in a spectrophotometer at 414 nm. Data were compared with negative (PBS buffer incubation) and positive (Triton X-100 0.1%) controls. Experiments were performed in triplicate, and the percent hemolysis was calculated as follows: (%) = (Asample − Abuffer)/(ATriton − Abuffer) × 100.

#### 2.5.5. Antimicrobial Activity

The antimicrobial activity was evaluated by using disk diffusion method. Clinical isolates of *Staphylococcus aureus*, *Coagulase Negative Staphylococcus*, *Klebsiella pneumoniae*, *Acinetobacter baumannii*, *Escherichia coli*, and *Enterobacter cloacae* were cultured in Blood Agar plates before the tests. The 0.5 McFarland inoculum of each strain were prepared in 0.75% saline solution. The inoculum was added in Mueller Hinton Agar plates, and discs of filter-paper were placed in the plates. Ten microliters of *M. philippina* venom (10 mg/mL) was pipetted in the discs. The results were interpreted by visual inspection, by observing the inhibition halo formed around the discs.

### 2.6. Statistical Analysis

Data were evaluated using GraphPad Prism to verify statistical differences between groups. One-way ANOVA test, followed by Tukey posttest, was applied, and differences were considered significant when *p* < 0.05.

## 3. Results

### 3.1. *Macrorhynchia philippina* Morphology

**Figure 2:**
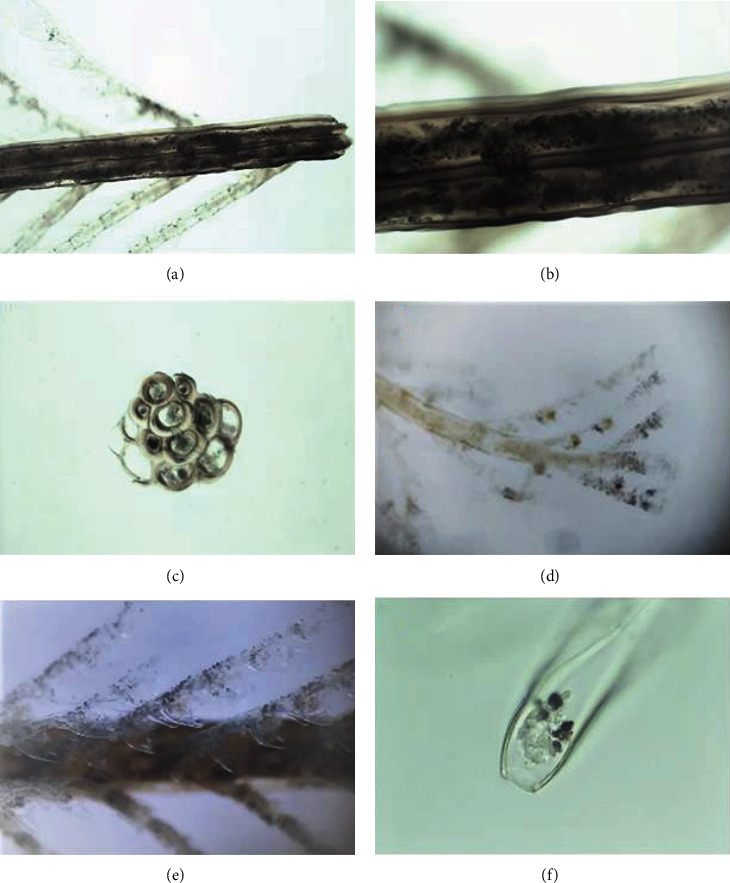
Light microscopy of *Macrorhynchia philippina* colony: (a) base of hydrocaulus and hydrocladium 40x; (b) detail of the hydrocaulus (middle of the structure) 100x; (c) hydrocaulus in a transversal view 40x; (d) hydrocaulus and hydrotheca 40x; (e) detail of hydrotheca in lateral view 100x; (f) detail of the final portion of hydrotheca 400x.

To study *M. philippina* morphology and understand its possible source of toxins, its colonies were firstly observed by light microscopy after fixation, without staining.  Figure 2 shows details of the hydrocaulus, axial structure, and its ramifications, the hydrocladiums.  Figure 2(a) depicts the longitudinal view of the basal portion of the hydrocaulus in the first plane, with the hydrocladiums behind. A detail of the hydrocaulus is shown in Figure 2(b), where it is possible to note the secretion inside the structure. In a transversal view (Figure 2(c)), it is possible to see that the hydrocaulus is composed of several canals, from the tip to the base of the structure (data not shown). The hydrocladium magnification is shown in Figure 2(d), where more ramifications are present in the tip of the structure, named hydrotheca. In this structure, the secretion is stored (black spots in the tip). The detail of hydrotheca, in a lateral view, is shown in Figures 2(e) and 2(f), where the content is ready to be released.

**Figure 3:**
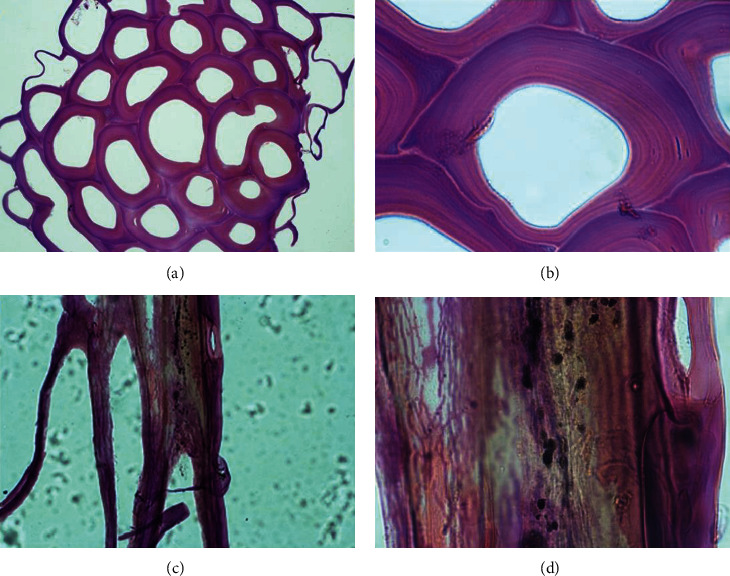
HE-stained *Macrorhynchia philippina* colony: (a) hydrocaulus in a transversal view 40x; (b) hydrocaulus in a transversal view 100x. Hydrocaulus in a longitudinal view 100x. Hydrocaulus in a longitudinal view 400x.

When the structures were stained with HE (Figures 3(a) and 3(b)), we could confirm the presence of canals, as observed before (Figure 2(c)). Apparently, the hydrocaulus is composed of structural proteins, such as keratin, that sustains all the structure (as a stalk). Nevertheless, it is possible to see a secretion inside the structure, where probably the secretory cells are present as well (Figures 3(c) and 3(d)).

**Figure 4:**
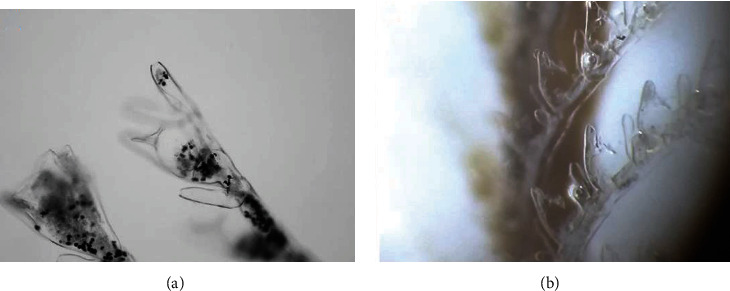
Analysis of the hydrotheca from *Macrorhynchia philippina* before (a) and after (b) the venom extraction.

To confirm the release of secretion after immersing the animal in methanol/acetic acid, we analyzed the morphology before and after the procedure. The secretion before the extraction was present in the hydrocladiums and hydrothecas (Figure 4(a)), where it is possible to see black spots. However, after 24 h immersion in methanol and acetic acid, no more secretions were visible in these structures, suggesting that its contents were completely released (Figure 4(b)). This content was analyzed by its composition and biological activity.

### 3.2. Biochemical Composition of the Venom

**Figure 5:**
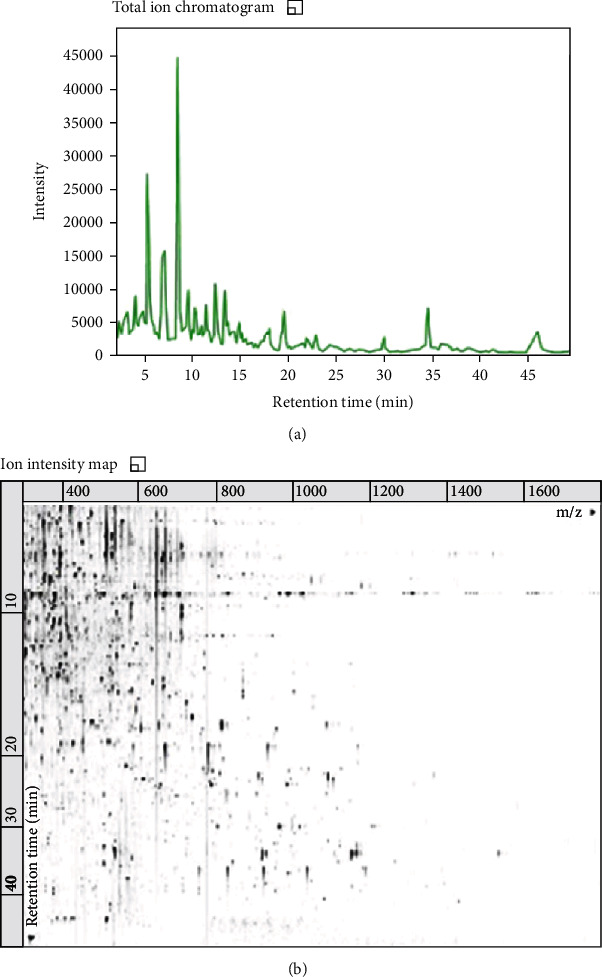
Biochemical profile of the methanolic *Macrorhynchia philippina* venom: (a) chromatographic profile showing peaks along the acetonitrile gradient; (b) ion intensity map, showed according to *m*/*z*, of molecules eluted by acetonitrile gradient.

The general biochemical profile of the *M. philippina* venom is shown in Figure 5(a), which shows peaks distributed along the acetonitrile gradient, being more abundant in the beginning, in a more hydrophilic elution. Moreover, according to the ion intensity map (Figure 5(b)), several ions between 100 and 1200 *m*/*z* were detected, being abundant in the 100-600 *m*/*z* region and between 0 and 15 minutes of gradient. This data indicates the abundance of low molecular mass compounds in the secretion.

Overall, 19 peptides were *de novo* sequenced (Table 1). When they were compared to the database (Blast analysis), no similarity with toxins from animal venoms were identified using BLAST search, indicating undescribed sequences. In addition, no proteins were visualized in the SDS-PAGE, even after stained with silver (data not shown) and only 5 proteins were identified by mass spectrometry, being 4 of them uncharacterized and 1 dynein heavy chain 12 from *Hydra vulgaris* (accession number tr|T2MAX4|T2MAX4_HYDVU).

On the other hand, 210 small molecules were identified, using the natural products and toxins database (Supplementary material (available here)). This data corroborated with the abundance of ions found around 500 *m*/*z*, shown in Figure 5(b). Ions and predicted molecular formula (obtained by the isotopic distribution) were compared to molecules previously described for hydroids, and then, we could find five known molecules, shown in Table 2.

### 3.3. Biological Activities

**Figure 6:**
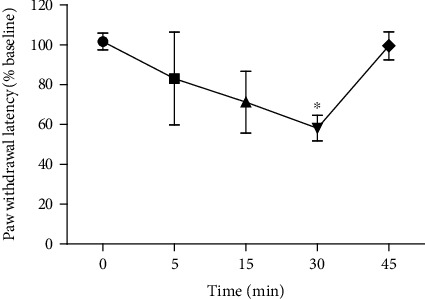
Mouse paw withdrawal latency (in % baseline) before and after the injection of *Macrorhynchia philippina* venom. ^∗^*p* < 0.05.

The *M. philippina* venom caused no hemolytic/cytotoxic activity in the tested doses, the same used for *in vivo* studies. However, in terms of its nociceptive activity, Figure 6 shows the time-course evaluation of paw withdrawal latency, in which it is possible to see that after 30 minutes of the secretion injection, a significant reduction of latency was observed, indicating a painful stimulus of the *M. philippina* venom.

**Figure 7:**
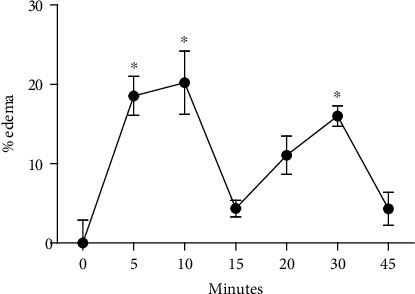
Mouse paw edema (%) before and after the injection of *Macrorhynchia philippina* venom. ^∗^*p* < 0.05.

Moreover, the venom induced a mild paw edema with two significant peaks—5/10 minutes and 30 minutes after injection. The edema was no longer visible after 45 minutes (Figure 7).

The 100 *μ*g *M. philippina* venom caused growth inhibition of *A. baumannii* and *K. pneumoniae*, observed as a halo around the discs. Although it is a high concentration, it was enough to identify an antibacterial effect, very important considering that these strains are resistant to antibiotics.

## 4. Discussion

Until now, the Phylum Cnidaria comprises more than 11000 species, according to the World Register of Marine Species (WorMS). They have one feature in common: the presence of nematocysts, a structure that releases a mixture of toxins, such as proteins and peptides, for prey capture and digestion, defense/predator repelling, locomotion, and intra-/interspecies spatial competition [17]. Although many cnidarians have this specialized structure, they may differ in shape and size according to the class. As they are full of venom, they can also cause human envenomation [15].

In general, human accidents caused by cnidarians are characterized by local repercussions and systemic, depending on the specie. Local symptoms are diverse, ranging from mild pain and itching to excruciating pain with necrosis and scarring, according to the amount of toxins injected and the venom-containing substances, cnidarian species, and its geographic distribution [18]. Cutaneous eruption can become persistent or generalized [17].

Accidents involving benthic hydroids are neglected, as such animals usually do not cause severe reactions and the venom action is apparently restricted to the site of the puncture [2]. According to Rifkin et al. [3], accidents with *M. philippina* are characterized by burning local pain, edema, erythema, and pruritus. Although these are mild symptoms, this specie has been recognized as health risk of population by envenomation in the Mediterranean region [4].

Similar patterns of envenomation have been related for other hydroids, for example *Nemalecium lighti* (*Haleciidae* family) that causes burning pain sensation, erythema, edema, and pruritus once in contact with the skin [19]. The Christmas-tree-hydroid *Pennaria disticha* also causes intense pain, burning sensation, erythema, and edema [20].

Although there are reports of envenomation, the biochemical nature of hydroids' venom is poorly explored, as the molecules are not easily accessed. Rifkin et al. [3] showed that the administration of vinegar triggered the discharge of the nematocysts (cells that harbor the venom) from *Lytocarpus philippinus*. Similarly, [21] used ethanol to induce nematocyst discharge to assess the venom content of the box jellyfish *Chironex fleckeri*.

The extraction showed here was confirmed by microscopy that enabled the visualization of empty structures and later corroborated by the biochemical and biological analysis. Moreover, the histology of colonies of *M. philippina* revealed a semirigid structure entirely filled with secretion, which is released in the tip (hydrotheca) [2].

Data from our group showed that methanol containing 0.1% acetic acid was enough to induce the extraction of molecules from *Chiropsalmus quadrumanus* and *Olindias sambaquiensis* [22, 23]. Indeed, methanol and acetic acid were enough to extract several small molecules from *M. philippina* with mild polarity that would not be dissolved in the sea water. The presence of higher masses, such as 1000 to 1500 *m*/*z* may correspond to peptides, which need to be investigated. Nevertheless, the abundance of molecules below 600 *m*/*z* is evident.

The biochemical composition of the most studied cnidarians has a protein nature. In general, hydrozoans have a predominance of phospholipases, cytotoxins, and hemolysins [24], whereas its jellyfishes have metallopeptidases, neurotoxins, and serine peptidases [9]. Also, the venom of *Tubastraea coccinea* was determined to harbor several neurotoxins, cytolysins, and dyshomeostasis [25]. On the other hand, the venom of *M. philippina* does not contain protein toxins, and the toxic effects were elicited by low molecular mass compounds. This data explains the lack of cytotoxic/hemolytic activity by *M. philippina* in opposite of similar cnidarians.

In terms of peptides, although 19 were identified herein, none of them is described in the literature. Peptides have been reported in the peptidome of the sea anemone *Phymanthus crucifer*, with several biological activities associated, which revealed 504 natural peptides secreted [26]. In *Hydra* and other cnidarians, peptides play an important role in the development as hormones and neurotransmitters, regulating reproduction, metamorphosis, muscle contraction, sensorial activity, among other crucial biological activities [27]. On the other hand, in the same phylum exists an important group of nonpeptidic toxins, well described for anthozoans [28].

Among the nonpeptidic toxins, we can list sarcophine-a, a cembranolide from *Sarcophyton glaucum*. This diterpene toxin was isolated from a hexane fraction and displayed muscarinic activities [29] and inhibits phosphofructokinase [30]. Regarding neurotransmission, lophotoxin is a furanocembranolide isolated by chloroform extraction from *Leptogorgia* sp. and has blocking activity on nicotinic acetylcholine receptors [31]. However, the most famous nonpeptidic marine toxin is the potent palytoxin, isolated by water/ethanol extraction from *Palythoa* sp. [32]. This toxin belongs to a family of polyhydroxylated (polyol) toxins that range between 2659 and 2680 Da, and blocks Na^+^/K^+^-ATPase channels as a toxic mechanism [33].

In *Millepora complanata* (Hydrozoa), a fraction of an aqueous extract had the presence of at least four nonpeptidic thermostable toxins that induced a potent vasoconstriction, hemolysis, and death in mice. Spectrometric analysis revealed that these toxins are polyoxygenated alkylbenzenes [34].

Here, we could find only five molecules already described from *M. philippina* among the 210 identified: isololiolide, Macrophilones and lytophilippines.

An isololiolide described by [35] had antitrypanosomal activity, without inducing cytotoxicity for mammalian cells. Cytotoxicity was observed by an isololiolide described by Vizetto-Duarte et al. [36], with apoptosis in tumor cell line. Zlotkowski et al. [37] showed that Macrophilone A increased intracellular reactive oxygen species levels and caused cytotoxicity in lung adenocarcinoma cells. Further studies showed that other Macrophilones caused cytotoxicity as well [38]. Lytophilippines were able to induce antibacterial, antiviral, and antitumor activities, besides displaying lethality in shrimps [39].

Antibacterial activity was identified here, against *A. baumannii* and *K. pneumoniae* strains, both gram-negative bacteria. *K. pneumoniae* is resistant to a broad spectrum of antibiotics and then causes serious health infection problems [40]. *A. baumannii* is another opportunistic bacterium, also resistant to antibiotics and frequently associated to respiratory infections [41]. The action of *M. philippina* venom on those strains indicates a different mechanism of bacterial death or growth inhibition, important as a chemical defense for the animal and, consequently, the maintenance of the specie in a hostile environment. Moreover, the discovery of new antibacterial molecules is extremely relevant for the clinics and biomedical field, considering the high incidence of antibiotic-resistant infections observed for several strains.

Considering that *M. philippina* accident causes pain and edema, we used animal models of mammals to evaluate these effects. We could observe a reduction of latency to pain behavior, indicating a hyperalgesia of the extract. The heat induces the activation of heat capsaicin-gated channel TRPV-1 by the release of inflammatory mediators, such as bradykinin, prostaglandins, chemokines, and ATP [42].

Besides activation of nociceptors, bradykinin is an inflammatory mediator that causes increase of vascular permeability, which increases the plasma flow to the extravascular compartment and causes edema. The action of such mediator is fast [43], explaining the effect after only 10 minutes of *M. philippina* venom injection.

Overall, the edema had two peaks—10 minutes and 30 minutes after the injection. This profile indicates the participation of more than one inflammatory mediator. Thus, besides bradykinin, other molecules might be acting to cause the inflammatory reaction.

Local inflammation is developed by the action of prostaglandins (PGs), histamine, serotonin, bradykinin, leukotrienes, and nitric oxide [44]. In the mouse edema examined herein, the fast inflammatory action (up to 30 minutes) indicates the release of stored mediators, such as histamine and serotonin. However, it is important to mention that the fast inflammatory effect summed to hyperalgesia is compatible with the envenomation profile of venom from *M. philippina*.

## 5. Conclusion

In conclusion, the *M. philippina* venom contains peptides and several low molecular mass compounds, able to cause fast inflammatory and nociceptive reactions, characteristic of envenomation, and antimicrobial activity, important for animal's defense.

## Figures and Tables

**Table 1:** Peptides identified in the *Macrorhynchia philippina* venom.

Sequence	ALC (%)	*m*/*z*	*z*	Mass
LPLYKNLH	85	499.3150	2	996.5756
VPPFLFEPV	81	522.7990	2	1043.5691
PMHPLFAYP	75	536.8000	2	1071.5212
LGSMCSNAVLGP	73	574.7790	2	1147.5366
NPGQPLYKGGLH	73	640.8870	2	1279.6672
HSCDCM	72	695.2530	1	694.1873
DPVDLDDGPPL	70	576.7980	2	1151.5347
DRPPLYKGGLH	70	626.8920	2	1251.6724
EEEEEEPDGN	68	588.7440	2	1175.4102
FDCYD	66	662.2050	1	661.2054
SSQFAFEEVEY	64	668.2630	2	1334.5667
MNDLPKPA	62	885.5470	1	884.4426
HAPLVVEGLVAVMKN	61	788.9920	2	1575.8806
MFAN	57	482.2047	1	481.1995
APPMVKYW	56	496.3000	2	990.4997
PVTLKKKC	53	458.7680	2	915.5576
CHAAPLCLTHLGVLVL	53	830.4670	2	1658.8999
PAGEPTWF	52	452.6725	2	903.4127
PETGALCVGAVSHC	51	672.3540	2	1342.6008

**Table 2:** Ions identified in the *Macrorhynchia philippina* venom previously described in the literature.

*m*/*z* found	*m*/*z* literature	Molecular formula	Compound
219.0320 [M+Na]+	219.0993 [M+Na]+	C_11_H_16_O_3_	Isololiolide
238.0390 [M+H]+	238.0645 [M+H]+	C_10_H_11_N_3_O_2_S	Macrophilone A
255.0383 [M+H]+	255.0428 [M+H]+	C_10_H_10_N_2_O_4_S	Macrophilone B
250.0344 [M+H]+	250.0646 [M+H]+	C_11_H_11_N_3_O_2_S	Macrophilone C
715.3977 [M+Na]+	715.3751 [M+Na]+	C_34_H_57_^35^ClO_12_	Lytophilippine A

## Data Availability

Data is available in supplementary information files.

## Supplementary Materials

Supplementary Materials Supplementary Table 1: low molecular mass compounds identified in the *M. philippina* venom.
