# Supplementary material for: Biochemical and Toxinological Characterization of Venom from Macrorhynchia philippina (Cnidaria, Hydrozoa)
Source: Biomed Res Int. 2022 May 17;2022:8170252. doi: 10.1155/2022/8170252 (PMC9129954; doi:10.1155/2022/8170252)
Supplement: Supplementary Materials — Supplementary Table 1: low molecular mass compounds identified in the M. philippina venom. [file 8170252.f1.docx]

**Supplementary table 1 – Low molecular mass compounds identified in the *M. philippina* venom.**

| **Compound** | **Compound ID** | **Formula** | **Score** | **Mass Error (ppm)** | **Description** |
| --- | --- | --- | --- | --- | --- |
| 2,01_423,1083m/z | CCMSLIB00000080278 | C23H18O8 | 37,8 | 1,94608E+14 | "MLS001048953-01![9-(1,3-benzodioxol-5-yl)-6,7-dimethoxy-1-oxo-3H-benzo[f][2]benzofuran-4-yl] acetate" |
| 2,01_324,1014m/z | CCMSLIB00000081585 | C16H15F2NO4 | 34,7 | -8,5142E+14 | Ethyl 1-cyclopropyl-6,7-difluoro-8-methoxy-4-oxo-1,4-dihydro-3-quinolinecarboxylate |
| 2,11_300,0782m/z | CCMSLIB00000085142 | C15H13N3O2S | 34,4 | -6,36358E+14 | MLS001201824-01!1-(6-Methoxy-benzothiazol-2-yl)-3-phenyl-urea |
| 2,11_300,0782m/z | CCMSLIB00000085711 | C10H13N5O4S | 35 | 7,08726E+14 | MLS002153781-01!Thioguanosine85-31-4 |
| 2,22_333,1296m/z | CCMSLIB00005436494 | C13H20N2O8 | 36,7 | 1,01415E+14 | Shinorine |
| 2,37_367,1656m/z | CCMSLIB00004680007 | C21H22N2O4 | 38,6 | 8,70106E+13 | Lundurine A |
| 2,37_475,1154m/z | CCMSLIB00000078012 | C22H24BrFN4O2 | 30,3 | 3,09736E+14 | Vandetanib (Zactima) |
| 2,37_310,0996m/z | CCMSLIB00004722014 | C17H15N3OS | 36,2 | -4,07538E+14 | MMV019189 |
| 2,50_341,0883m/z | CCMSLIB00000846347 | C15H16O9 | 37,7 | 4,59605E+14 | NCGC00094874-06!7-hydroxy-6-[(2S,3R,4S,5S,6R)-3,4,5-trihydroxy-6-(hydroxymethyl)oxan-2-yl]oxychromen-2-one |
| 2,50_341,0883m/z | VF-NPL-QTOF006527 | C15H16O9 | 37,7 | 4,59605E+14 | Aesculin |
| 2,65_301,1531m/z | CCMSLIB00001058266 | C17H20N2O3 | 35,2 | -5,26207E+14 | Pesticide3_Bifenazate_C17H20N2O3_1-Methylethyl 2-(4-methoxybiphenyl-3-yl)hydrazinecarboxylate |
| 3,26_367,1654m/z | CCMSLIB00004680007 | C21H22N2O4 | 39,2 | 4,87411E+14 | Lundurine A |
| 3,26_451,1415m/z | CCMSLIB00000840431 | C25H22O8 | 33,6 | 6,16616E+14 | Galtamycinone |
| 3,26_451,1415m/z | CCMSLIB00000850428 | C18H26O13 | 34,4 | -6,88156E+14 | NCGC00384661-01_C18H26O13_Cyclopenta[c]pyran-4,7-dicarboxylic acid, 1-(beta-D-glucopyranosyloxy)-1,4a,5,6,7,7a-hexahydro-5,6-dihydroxy-, dimethyl ester, (1S,4aS,6R,7R,7aS)- |
| 3,26_451,1415m/z | VF-NPL-QTOF008062 | C25H22O8 | 33,6 | 6,16616E+14 | Irigenin, 7-Benzyl Ether |
| 3,26_389,1481m/z | CCMSLIB00000078768 | C17H24O10 | 37 | 9,97308E+14 | NCGC00160232-01!VERBENALIN |
| 3,26_389,1481m/z | CCMSLIB00000848586 | C17H24O10 | 37 | 9,97308E+14 | NCGC00380093-01!methyl 3-ethenyl-4-(2-oxoethyl)-2-[3,4,5-trihydroxy-6-(hydroxymethyl)oxan-2-yl]oxy-3,4-dihydro-2H-pyran-5-carboxylate |
| 3,26_389,1481m/z | CCMSLIB00000849841 | C17H24O10 | 37 | 9,97308E+14 | NCGC00381280-01_C17H24O10_Phenyl 6-O-[(2R,3R,4R)-3,4-dihydroxy-4-(hydroxymethyl)tetrahydro-2-furanyl]-beta-D-glucopyranoside |
| 3,26_389,1481m/z | CCMSLIB00000852136 | C17H24O10 | 37 | 9,97308E+14 | NCGC00385262-01_C17H24O10_Cyclopenta[c]pyran-4-carboxylic acid, 1-(beta-D-glucopyranosyloxy)-1,4a,5,6,7,7a-hexahydro-7-methyl-5-oxo-, methyl ester, (1S,7R)- |
| 3,26_389,1481m/z | CCMSLIB00000854324 | C17H24O10 | 37 | 9,97308E+14 | NCGC00346662-04_C17H24O10_Methyl (1S,4aS,7aS)-1-(beta-D-glucopyranosyloxy)-7-(hydroxymethyl)-1,4a,5,7a-tetrahydrocyclopenta[c]pyran-4-carboxylate |
| 3,26_389,1481m/z | VF-NPL-QTOF007785 | C17H24O10 | 37 | 9,97308E+14 | geniposide |
| 3,26_352,0782m/z | CCMSLIB00000085668 | C17H18ClNO3S | 33,9 | 3,9233E+14 | MLS001076075-01!402-71-1 |
| 3,44_321,0974m/z | CCMSLIB00000846552 | C16H16O7 | 31,5 | 1,6809E+14 | NCGC00169879-02!2-hydroxy-6-[2-hydroxy-6-(hydroxymethyl)-4-methoxyphenoxy]-4-methylbenzoic acid |
| 3,66_337,0931m/z | CCMSLIB00000845375 | C16H16O8 | 32,3 | 3,88707E+14 | NCGC00380391-01!2-(4a,9,10a-trihydroxy-1-methyl-5,10-dioxo-3,4-dihydro-1H-benzo[g]isochromen-3-yl)acetic acid |
| 3,66_337,0931m/z | VF-NPL-QTOF002761 | C16H16O8 | 32,3 | 3,88707E+14 | 2-(4a,9,10a-trihydroxy-1-methyl-5,10-dioxo-3,4-dihydro-1H-benzo[g]isochromen-3-yl)acetic acid |
| 3,98_313,0975m/z | CCMSLIB00004722050 | C14H18Cl2N4 | 30,6 | -2,00944E+14 | MMV689060 |
| 4,07_382,0804m/z | CCMSLIB00000078076 | C17H14F3N3O2S | 36,6 | -7,19383E+14 | Celecoxib |
| 4,07_529,1391m/z | CCMSLIB00000847572 | C26H24O12 | 35,9 | 9,54358E+14 | NCGC00380722-01![(2S,3R,4S,5S,6R)-4,5-dihydroxy-6-(hydroxymethyl)-2-[2,4,6-trihydroxy-3-(4-hydroxybenzoyl)phenyl]oxan-3-yl] 4-hydroxybenzoate |
| 4,07_529,1391m/z | VF-NPL-QTOF005919 | C26H24O12 | 35,9 | 9,54358E+14 | [(2S,3R,4S,5S,6R)-4,5-dihydroxy-6-(hydroxymethyl)-2-[2,4,6-trihydroxy-3-(4-hydroxybenzoyl)phenyl]oxan-3-yl] 4-hydroxybenzoate |
| 4,52_310,0986m/z | CCMSLIB00004722119 | C17H15N3OS | 33,9 | -7,4391E+14 | MMV019189 |
| 4,52_539,1814m/z | CCMSLIB00004722185 | C36H26O5 | 35,9 | -7,26609E+14 | Selaginpulvilin K |
| 5,24_380,1438m/z | CCMSLIB00000079651 | C21H21N3O2S | 37 | 2,90151E+14 | MLS001076858-01! |
| 5,24_412,1347m/z | CCMSLIB00001058499 | C23H22ClNO4 | 31,7 | 8,86609E+14 | Pesticide4_Mandipropamid_C23H22ClNO4_2-(4-Chlorophenyl)-N-{2-[3-methoxy-4-(2-propyn-1-yloxy)phenyl]ethyl}-2-(2-propyn-1-yloxy)acetamide |
| 5,34_331,0981m/z | CCMSLIB00000085077 | C16H15ClN4O2 | 33,1 | 7,34631E+14 | MLS000860039-01!8-(3-Chlorostyryl)caffeine |
| 5,34_382,0801m/z | CCMSLIB00000078076 | C17H14F3N3O2S | 36,1 | -8,00931E+14 | Celecoxib |
| 5,34_382,0801m/z | CCMSLIB00000084890 | C17H14F3N3O2S | 36,1 | -8,00931E+14 | MLS001195656-01!Celecoxib169590-42-5 |
| 5,59_318,2048m/z | CCMSLIB00000078488 | C19H27NO3 | 34,9 | -4,97866E+13 | Nateglinide (Starlix) |
| 5,96_308,0859m/z | CCMSLIB00000079216 | C14H16ClN4S | 33,4 | 6,28194E+14 | 3-(1H-imidazol-5-yl)propyl N'-[(4-chlorophenyl)methyl]carbamimidothioate |
| 6,13_310,0978m/z | CCMSLIB00004722014 | C17H15N3OS | 24,8 | -9,83354E+14 | MMV019189 |
| 6,36_542,1678m/z | CCMSLIB00000080433 | C30H25N2O8 | 38,4 | -1,10134E+14 | MLS001158027-01!3-(5-hydroxy-1H-indol-3-yl)-2-[2-[4-(4-methoxyphenyl)-2-oxochromen-7-yl]oxypropanoylamino]propanoic acid |
| 6,73_321,0980m/z | CCMSLIB00000846179 | C16H16O7 | 35,7 | 3,37954E+14 | NCGC00169879-02!2-hydroxy-6-[2-hydroxy-6-(hydroxymethyl)-4-methoxyphenoxy]-4-methylbenzoic acid |
| 6,89_454,1096m/z | CCMSLIB00000080268 | C23H19NO9 | 37,1 | -7,99114E+14 | "MLS001157775-01!(2S)-1-[2-[[(2Z)-2-(1,3-benzodioxol-5-ylmethylidene)-3-oxo-1-benzofuran-6-yl]oxy]acetyl]-4-hydroxypyrrolidine-2-carboxylic acid" |
| 7,27_355,0755m/z | CCMSLIB00000078629 | C16H21BrNO3 | 29,4 | -6,30171E+14 | Homatropine Bromide |
| 7,27_355,0755m/z | CCMSLIB00000084780 | C16H21BrNO3 | 29,4 | -6,30171E+14 | MLS002172468-01!Dihydro-beta-erythroidine hydrobromide29734-68-7 |
| 7,27_355,0755m/z | CCMSLIB00000085346 | C16H21BrNO3 | 29,4 | -6,30171E+14 | "MLS002154189-01!Homatropine hydrobromide (R,S)51-56-9" |
| 7,69_368,1070m/z | CCMSLIB00000078459 | C17H18N3NaO3S | 33,7 | 8,26798E+14 | Esomeprazole sodium (Nexium) |
| 7,69_368,1070m/z | CCMSLIB00001058545 | C18H16F3NO4 | 34,2 | -9,40002E+14 | Pesticide4_Picoxystrobin_C18H16F3NO4_Acanto |
| 8,49_364,1113m/z | CCMSLIB00000079024 | C17H18FN3O3S | 37,4 | -3,47763E+14 | rufloxacin monohydrochloride |
| 8,49_615,1923m/z | CCMSLIB00000851055 | C27H34O16 | 34,2 | 5,67634E+14 | NCGC00169372-02_C27H34O16_2-(beta-D-Glucopyranosyloxy)benzyl 3-(hexopyranosyloxy)-6-hydroxy-2-methoxybenzoate |
| 8,49_615,1923m/z | VF-NPL-QTOF006140 | C27H34O16 | 34,2 | 5,67634E+14 | [2-[(2S,3R,4S,5S,6R)-3,4,5-trihydroxy-6-(hydroxymethyl)oxan-2-yl]oxyphenyl]methyl 6-hydroxy-2-methoxy-3-[3,4,5-trihydroxy-6-(hydroxymethyl)oxan-2-yl]oxybenzoate |
| 8,49_380,1444m/z | CCMSLIB00000079651 | C21H21N3O2S | 37,1 | 4,5474E+14 | MLS001076858-01! |
| 8,49_304,0886m/z | CCMSLIB00000077052 | C14H19Cl2NO2 | 31,4 | 6,86889E+14 | 305-03-3 |
| 8,94_368,1068m/z | CCMSLIB00000078459 | C17H18N3NaO3S | 36,8 | 7,85177E+14 | Esomeprazole sodium (Nexium) |
| 8,94_368,1068m/z | CCMSLIB00001058545 | C18H16F3NO4 | 37,3 | -9,81623E+14 | Pesticide4_Picoxystrobin_C18H16F3NO4_Acanto |
| 8,94_311,0895m/z | CCMSLIB00000085700 | C15H21BrNO | 28,8 | 4,91426E+14 | "MLS002153407-01!cis-8-Hydroxy-3-(n-propyl)-1,2,3a,4,5,9b-hexahydro-1H-benz[e]indole hydrobromide" |
| 9,58_378,1274m/z | CCMSLIB00000478076 | C18H23N3O2S2 | 32,8 | -8,06673E+14 | Polanrazine B_120170 |
| 9,74_307,1050m/z | CCMSLIB00000084778 | C18H14N2O3 | 34,6 | -8,75056E+14 | MLS002153157-01!Tyrphostin AG 528 |
| 9,88_542,1671m/z | CCMSLIB00000080433 | C30H25N2O8 | 33,5 | -2,35984E+14 | MLS001158027-01!3-(5-hydroxy-1H-indol-3-yl)-2-[2-[4-(4-methoxyphenyl)-2-oxochromen-7-yl]oxypropanoylamino]propanoic acid |
| 9,88_433,0510m/z | CCMSLIB00000086082 | C18H20Cl4N4 | 25,6 | -1,23456E+14 | MLS000860080-01!NBI 27914 |
| 10,02_367,0691m/z | CCMSLIB00000848584 | C16H14O10 | 36,7 | 8,41068E+14 | NCGC00380094-01!4-[(E)-3-(3,4-dihydroxyphenyl)prop-2-enoyl]oxy-3-hydroxy-3,4-dihydro-2H-pyran-2,6-dicarboxylic acid |
| 10,14_366,1307m/z | CCMSLIB00000078762 | C21H19NO5 | 33,9 | -7,81628E+14 | NCGC00160212-01!THALICSINE |
| 10,14_366,1307m/z | CCMSLIB00005436043 | C21H19NO5 | 33,9 | -7,81628E+14 | 11-methoxyduguesuramine |
| 10,26_334,1545m/z | CCMSLIB00000078932 | C17H20FN3O3 | 38 | -4,98468E+14 | PEFLOXACIN MESYLATE |
| 10,26_334,1545m/z | CCMSLIB00000085762 | C17H22ClN4O | 34 | -3,01653E+14 | MLS001146964-01! |
| 10,26_334,1545m/z | CCMSLIB00000846661 | C20H19N3O2 | 39 | -1,55417E+14 | NCGC00347762-02!3-benzyl-6-(1H-indol-3-ylmethyl)piperazine-2,5-dione |
| 10,40_394,2023m/z | CCMSLIB00000079703 | C22H25N4O3 | 36,8 | 5,90266E+14 | MLS001142707-01! |
| 10,40_316,2259m/z | CCMSLIB00000846683 | C20H29NO2 | 37,8 | -3,71597E+14 | NCGC00380344-01!(2E,4E)-N-[2-(4-hydroxyphenyl)ethyl]dodeca-2,4-dienamide |
| 10,95_414,0967m/z | CCMSLIB00000085066 | C17H16ClF6N2O | 34,5 | 9,48555E+14 | MLS001332559-01!Mefloquine hydrochloride51773-92-3 |
| 10,95_501,1392m/z | VF-NPL-QTOF007016 | C25H24O11 | 38,5 | 1,53827E+14 | Catechin Pentaacetate |
| 10,95_501,1392m/z | VF-NPL-QTOF007595 | C25H24O11 | 38,5 | 1,53827E+14 | Epicatechin Pentaacetate |
| 11,10_465,1026m/z | CCMSLIB00000077057 | C21H21ClN2O8 | 31,9 | -7,08968E+14 | Demeclocycline |
| 11,10_465,1026m/z | CCMSLIB00000077231 | C21H20O12 | 37,1 | -2,65132E+14 | Isoquercitrin |
| 11,10_465,1026m/z | CCMSLIB00000078067 | C21H16F4N4O2S | 35,8 | 5,04937E+14 | MDV3100 (Enzalutamide) |
| 11,10_465,1026m/z | CCMSLIB00000845303 | C21H20O12 | 37,1 | -2,65132E+12 | NCGC00168902-03!2-(3,4-dihydroxyphenyl)-5,7-dihydroxy-3-[(2S,3R,4S,5R,6R)-3,4,5-trihydroxy-6-(hydroxymethyl)oxan-2-yl]oxychromen-4-one |
| 11,10_465,1026m/z | CCMSLIB00004678825 | C21H20O12 | 37,1 | -2,65132E+12 | quercetin-3-O-hexoside |
| 11,10_465,1026m/z | CCMSLIB00004684183 | C21H20O12 | 37,1 | -2,65132E+12 | Myricitrin |
| 11,10_465,1026m/z | CCMSLIB00004684184 | C21H20O12 | 37,1 | -2,65132E+12 | Spiraeoside |
| 11,10_465,1026m/z | CCMSLIB00004684186 | C21H20O12 | 37,1 | -2,65132E+12 | Isoquercitin |
| 11,10_465,1026m/z | VF-NPL-QTOF008017 | C21H20O12 | 37,1 | -2,65132E+12 | hyperoside |
| 11,10_461,1273m/z | CCMSLIB00000078370 | C25H27Cl3N2 | 26,2 | -8,52211E+14 | Meclizine dihydrochloride |
| 11,10_461,1273m/z | CCMSLIB00000085308 | C22H22ClKN6O | 31,6 | 4,32935E+14 | MLS001165733-01!losartan |
| 11,27_367,0755m/z | CCMSLIB00000085961 | C17H21BrNO3 | 27 | -6,05248E+14 | MLS001401401-01!Galanthamine |
| 11,57_341,1023m/z | CCMSLIB00000080263 | C19H16O6 | 35,8 | 9,96953E+14 | MLS002473152-01![6-methoxy-3-(4-methoxyphenyl)-4-oxochromen-7-yl] acetate |
| 11,73_310,0997m/z | CCMSLIB00004722014 | C17H15N3OS | 37,3 | -3,86528E+14 | MMV019189 |
| 12,27_349,1987m/z | CCMSLIB00000080473 | C20H28O5 | 36,5 | -6,36158E+14 | MLS001143521-01! |
| 12,27_349,1987m/z | CCMSLIB00000849542 | C20H28O5 | 36,5 | -6,36158E+14 | NCGC00381207-01_C20H28O5_2(5H)-Furanone, 3-[2-[(1R,4aS,5R,6R,8aS)-decahydro-6-hydroxy-5-(hydroxymethyl)-5,8a-dimethyl-2-methylene-1-naphthalenyl]acetyl]- |
| 12,27_349,1987m/z | CCMSLIB00000851223 | C20H28O5 | 36,5 | -6,36158E+14 | NCGC00380833-01_C20H28O5_Gibbane-1,10-dicarboxylic acid, 2-hydroxy-1,4a-dimethyl-8-methylene-, (1alpha,2beta,4aalpha,4bbeta,10beta)- |
| 12,27_349,1987m/z | CCMSLIB00000852219 | C20H28O5 | 36,5 | -6,36158E+14 | NCGC00380426-01_C20H28O5_7,10-Methano-1H-benzocycloundecene-4-carboxylic acid, 2,3,4,4a,5,6,9,10,11,12,13,13a-dodecahydro-14-hydroxy-4,13a-dimethyl-9,13-dioxo-, methyl ester, (4R,4aS,10R,13aR)- |
| 12,27_349,1987m/z | CCMSLIB00000852834 | C20H28O5 | 36,5 | -6,36158E+14 | NCGC00380913-01_C20H28O5_2(3H)-Furanone, 5-[4-[1-(acetyloxy)-4-oxo-5-[(2Z)-2-penten-1-yl]-2-cyclopenten-1-yl]butyl]dihydro- |
| 12,27_349,1987m/z | CCMSLIB00000854881 | C20H28O5 | 36,5 | -6,36158E+14 | NCGC00380451-01_C20H28O5_2-(3-Furyl)-7,8-dihydroxy-6a,7,10b-trimethyldodecahydro-4H-benzo[f]isochromen-4-one |
| 12,27_349,1987m/z | CCMSLIB00005435626 | C20H28O5 | 36,5 | -6,36158E+14 | Euphactin G from S.guyanensis |
| 12,27_349,1987m/z | CCMSLIB00005435627 | C20H28O5 | 36,5 | -6,36158E+14 | Euphoractine P from S.guyanensis |
| 12,27_349,1987m/z | CCMSLIB00005435631 | C20H28O5 | 36,5 | -6,36158E+14 | Euphoractine S from S.guyanensis |
| 12,27_349,1987m/z | CCMSLIB00005435632 | C20H28O5 | 36,5 | -6,36158E+14 | Euphoractine T from S.guyanensis |
| 12,27_349,1987m/z | CCMSLIB00005435633 | C20H28O5 | 36,5 | -6,36158E+14 | Euphoractine U from S.guyanensis |
| 12,27_349,1987m/z | CCMSLIB00005435634 | C20H28O5 | 36,5 | -6,36158E+14 | Euphoractine V from S.guyanensis |
| 12,27_349,1987m/z | CCMSLIB00005435638 | C20H28O5 | 36,5 | -6,36158E+14 | Euphactin G from S.guyanensis |
| 12,40_363,1783m/z | CCMSLIB00000079066 | C20H26O6 | 34,6 | -5,22865E+14 | SECOISOLARICIRESINOL |
| 12,40_363,1783m/z | CCMSLIB00000851973 | C20H26O6 | 34,6 | -5,22865E+14 | NCGC00380706-01_C20H26O6_(2'R,3R,4'R,4a'R,5S,8a'S)-5-(3-Furyl)-4'-hydroxy-4a',5'-bis(hydroxymethyl)-2'-methyl-3',4,4',4a',5,7',8',8a'-octahydro-2'H-spiro[furan-3,1'-naphthalen]-2-one |
| 12,40_363,1783m/z | CCMSLIB00000852530 | C20H26O6 | 34,6 | -5,22865E+13 | NCGC00380263-01_C20H26O6_2-Butenoic acid, 2-methyl-, decahydro-3',5'a-dimethyl-2'-oxospiro[oxirane-2,8'(2'H)-oxireno[6,7]naphtho[1,2-b]furan]-6'-yl ester, (2Z)- |
| 12,40_363,1783m/z | CCMSLIB00000854021 | C20H26O6 | 34,6 | -5,22865E+13 | NCGC00169747-02_C20H26O6_Spiro[furan-3(2H),6'(8'H)-[2H,3H]naphth[1,8a-b]oxet]-2-one, 5-(3-furanyl)octahydro-3'-hydroxy-9'a-(hydroxymethyl)-5'-methyl-, (2a'R,3R,3'R,5S,5'R,6a'S,9a'R)- |
| 12,40_363,1783m/z | CCMSLIB00000854500 | C20H26O6 | 34,6 | -5,22865E+13 | NCGC00381455-01_C20H26O6_Propanoic acid, 2-methyl-, (3aS,4S,5S,6E,10E,11aR)-6-formyl-2,3,3a,4,5,8,9,11a-octahydro-5-methoxy-10-methyl-3-methylene-2-oxocyclodeca[b]furan-4-yl ester |
| 12,40_363,1783m/z | CCMSLIB00000855892 | C20H26O6 | 34,6 | -5,22865E+13 | NCGC00384904-01_C20H26O6_(3R,3'R,3a'R,5S,6a'S,8'R,10'R,10a'R)-5-(3-Furyl)-3',10'-dihydroxy-8'-methyldecahydro-8'H-spiro[furan-3,7'-naphtho[1,8a-c]furan]-2-one |
| 12,40_363,1783m/z | VF-NPL-QTOF002045 | C20H26O6 | 34,6 | -5,22865E+13 | (E)-8-(4-hydroxy-6-methoxy-7-methyl-3-oxo-1H-2-benzofuran-5-yl)-2,6-dimethyloct-6-enoic acid |
| 12,40_363,1783m/z | VF-NPL-QTOF002584 | C20H26O6 | 34,6 | -5,22865E+13 | 2,3-bis[(4-hydroxy-3-methoxyphenyl)methyl]butane-1,4-diol |
| 12,40_363,1783m/z | VF-NPL-QTOF004590 | C20H26O6 | 34,6 | -5,22865E+13 | 5-hydroxy-7-[2-(2-hydroxy-5-oxo-2H-furan-3-yl)ethyl]-7,8-dimethyl-5,6,6a,8,9,10-hexahydro-1H-benzo[d][2]benzofuran-3-one |
| 12,40_363,1783m/z | VF-NPL-QTOF005989 | C20H26O6 | 34,6 | -5,22865E+13 | [(3aS,4S,5S,6E,10E,11aR)-6-formyl-5-methoxy-10-methyl-3-methylidene-2-oxo-3a,4,5,8,9,11a-hexahydrocyclodeca[b]furan-4-yl] 2-methylpropanoate |
| 12,97_452,1692m/z | CCMSLIB00000080139 | C25H25NO7 | 38,4 | -2,68474E+14 | "MLS001157850-01!(2S)-2-phenyl-2-[[2-[(4,8,8-trimethyl-2-oxo-9,10-dihydropyrano[2,3-h]chromen-5-yl)oxy]acetyl]amino]acetic acid" |
| 12,97_452,1692m/z | CCMSLIB00000085564 | C24H24FN4O2S | 37,7 | 3,30402E+14 | "MLS002172448-01!LY-367,265" |
| 13,14_501,1320m/z | CCMSLIB00000086151 | C21H27ClN3O7S | 30,6 | -2,22293E+13 | MLS002153801-01!Bacampicillin hydrochloride37661-08-8 |
| 13,38_394,2022m/z | CCMSLIB00000079996 | C22H25N4O3 | 36,8 | 5,76549E+14 | MLS001141002-01! |
| 13,38_412,1348m/z | CCMSLIB00001058499 | C23H22ClNO4 | 34,7 | 9,13436E+14 | Pesticide4_Mandipropamid_C23H22ClNO4_2-(4-Chlorophenyl)-N-{2-[3-methoxy-4-(2-propyn-1-yloxy)phenyl]ethyl}-2-(2-propyn-1-yloxy)acetamide |
| 13,38_349,0943m/z | CCMSLIB00000079632 | C17H16O8 | 37,3 | 7,15021E+14 | MLS000863583-01!2-hydroxy-6-(4-hydroxy-2-methoxy-6-methoxycarbonylphenoxy)-4-methylbenzoic acid |
| 13,38_349,0943m/z | CCMSLIB00000478054 | C17H16O8 | 37,3 | 7,15021E+14 | Asterric acid_120092 |
| 13,38_349,0943m/z | CCMSLIB00000478445 | C17H16O8 | 37,3 | 7,15021E+14 | Hydroxysulochrin_120241 |
| 13,38_349,0943m/z | CCMSLIB00000848011 | C17H16O8 | 37,3 | 7,15021E+14 | NCGC00180411-02!2-hydroxy-6-(4-hydroxy-2-methoxy-6-methoxycarbonylphenoxy)-4-methylbenzoic acid |
| 13,38_349,0943m/z | CCMSLIB00000848539 | C17H16O8 | 37,3 | 7,15021E+14 | NCGC00381013-01!5-hydroxy-2-(3-hydroxy-2-methoxycarbonyl-5-methylphenoxy)-3-methoxybenzoic acid |
| 13,38_349,0943m/z | VF-NPL-QTOF006781 | C17H16O8 | 37,3 | 7,15021E+14 | asterric acid |
| 13,52_335,0778m/z | CCMSLIB00000848785 | C16H14O8 | 35,4 | 4,90574E+14 | NCGC00385178-01!(2S,3S)-3,5,7-trihydroxy-6-methyl-2-(3,4,5-trihydroxyphenyl)-2,3-dihydrochromen-4-one |
| 13,52_335,0778m/z | CCMSLIB00004751244 | C16H14O8 | 35,4 | 4,90574E+14 | Diploschistesic acid |
| 13,52_335,0778m/z | CCMSLIB00004751434 | C16H14O8 | 35,4 | 4,90574E+14 | Methoxyhaemoventosine |
| 13,52_335,0778m/z | VF-NPL-QTOF001150 | C16H14O8 | 35,4 | 4,90574E+14 | (2S,3S)-3,5,7-trihydroxy-6-methyl-2-(3,4,5-trihydroxyphenyl)-2,3-dihydrochromen-4-one |
| 13,69_304,0886m/z | CCMSLIB00000077052 | C14H19Cl2NO2 | 29,6 | 6,82671E+14 | 305-03-3 |
| 14,16_439,1245m/z | CCMSLIB00000845589 | C20H22O11 | 35,1 | 2,22984E+14 | NCGC00385022-01![3-hydroxy-4-[(2S,3R,4S,5S,6R)-3,4,5-trihydroxy-6-(hydroxymethyl)oxan-2-yl]oxyphenyl]methyl 3,4-dihydroxybenzoate |
| 14,16_393,2261m/z | CCMSLIB00000845032 | C22H32O6 | 34,8 | -2,80312E+14 | NCGC00385669-01!(E)-3-(acetyloxymethyl)-5-(2-formyl-4-hydroxy-5,5,8a-trimethyl-1,4,4a,6,7,8-hexahydronaphthalen-1-yl)pent-2-enoic acid |
| 14,16_393,2261m/z | CCMSLIB00000851967 | C22H32O6 | 34,8 | -2,80312E+13 | NCGC00169206-02_C22H32O6_1-Naphthalenecarboxylic acid, 1,2,3,4,4a,5,8,8a-octahydro-1,4a,6-trimethyl-5-[(2,3,5-trihydroxy-4-methylene-7-oxabicyclo[4.1.0]hept-1-yl)methyl]- |
| 14,16_393,2261m/z | CCMSLIB00000855808 | C22H32O6 | 34,8 | -2,80312E+13 | NCGC00180384-03_C22H32O6_(1S,2R,4aR,8aR)-1-Acetoxy-7-isopropylidene-1,4a-dimethyl-6-oxodecahydro-2-naphthalenyl 2,3-dimethyl-2-oxiranecarboxylate |
| 14,45_306,1695m/z | CCMSLIB00000079032 | C17H23NO4 | 39,1 | -1,50541E+14 | CETRAXATE HCl |
| 14,45_416,2455m/z | CCMSLIB00005463672 | C24H33NO5 | 37,2 | 5,57339E+13 | Smenospongin C |
| 14,87_361,0900m/z | CCMSLIB00000078853 | C18H16O8 | 35 | -4,94548E+13 | Rosmarinic acid |
| 14,87_361,0900m/z | CCMSLIB00000080526 | C18H16O8 | 35 | -4,94548E+14 | "MLS000876977-01!5,8-dihydroxy-2-(4-hydroxy-3-methoxyphenyl)-6,7-dimethoxychromen-4-one" |
| 14,87_361,0900m/z | CCMSLIB00000080568 | C18H16O8 | 35 | -4,94548E+14 | "MLS001049145-01!2-(2,6-dihydroxyphenyl)-5-hydroxy-6,7,8-trimethoxychromen-4-one" |
| 14,87_361,0900m/z | CCMSLIB00000845638 | C18H16O8 | 35 | -4,94548E+14 | NCGC00180783-02!5,7-dihydroxy-2-(4-hydroxy-3-methoxyphenyl)-3,6-dimethoxychromen-4-one |
| 14,87_361,0900m/z | CCMSLIB00000847481 | C18H16O8 | 35 | -4,94548E+13 | NCGC00179783-02![(2R,3R)-2-(3,4-dihydroxyphenyl)-5-hydroxy-7-methoxy-4-oxo-2,3-dihydrochromen-3-yl] acetate |
| 14,87_361,0900m/z | CCMSLIB00000848000 | C18H16O8 | 35 | -4,94548E+14 | NCGC00384647-01!5,7-dihydroxy-3-(3-hydroxy-4,5-dimethoxyphenyl)-6-methoxychromen-4-one |
| 14,87_361,0900m/z | CCMSLIB00000848806 | C18H16O8 | 35 | -4,94548E+14 | NCGC00169741-02!2-(3,4-dihydroxyphenyl)-5-hydroxy-3,6,7-trimethoxychromen-4-one |
| 14,87_361,0900m/z | VF-NPL-QTOF003793 | C18H16O8 | 35 | -4,94548E+13 | 3-O-acetylpadmatin |
| 14,87_361,0900m/z | VF-NPL-QTOF008054 | C18H16O8 | 35 | -4,94548E+14 | IRIGENIN |
| 14,87_361,0900m/z | VF-NPL-QTOF008165 | C18H16O8 | 35 | -4,94548E+14 | jaceidin |
| 14,87_361,0900m/z | VF-NPL-QTOF009533 | C18H16O8 | 35 | -4,94548E+13 | rosmarinic acid |
| 14,87_350,1323m/z | CCMSLIB00000085403 | C19H23Cl2N2 | 28,5 | 3,34212E+14 | MLS001074205-01!17321-77-6 |
| 14,87_350,1323m/z | CCMSLIB00000085498 | C19H23Cl2N2 | 28,5 | 3,34212E+14 | MLS000028607-01! |
| 15,07_362,1327m/z | CCMSLIB00000078149 | C17H19N3O6 | 37,3 | -5,38208E+14 | Varenicline tartrate |
| 15,07_581,1807m/z | CCMSLIB00000079735 | C27H32O14 | 36,8 | -9,99471E+14 | MLS001143533-01! |
| 15,07_581,1807m/z | CCMSLIB00000084948 | C27H32O14 | 36,8 | -9,99471E+14 | MLS001335956-01!Naringin10236-47-2 |
| 15,07_581,1807m/z | CCMSLIB00000574565 | C27H32O14 | 36,8 | -9,99471E+14 | Cascaroside A |
| 15,07_581,1807m/z | CCMSLIB00000574567 | C27H32O14 | 36,8 | -9,99471E+14 | Cascaroside B |
| 15,07_581,1807m/z | CCMSLIB00000845095 | C27H32O14 | 36,8 | -9,99471E+14 | NCGC00169359-03!5-hydroxy-2-(4-hydroxyphenyl)-7-[(2S,3R,4S,5S,6R)-3,4,5-trihydroxy-6-[[(2R,3R,4R,5R,6S)-3,4,5-trihydroxy-6-methyloxan-2-yl]oxymethyl]oxan-2-yl]oxy-2,3-dihydrochromen-4-one |
| 15,07_581,1807m/z | CCMSLIB00000845182 | C27H32O14 | 36,8 | -9,99471E+14 | NCGC00380864-01!7-[4,5-dihydroxy-6-(hydroxymethyl)-3-[(2S,3R,4R,5R,6S)-3,4,5-trihydroxy-6-methyloxan-2-yl]oxyoxan-2-yl]oxy-5-hydroxy-2-(4-hydroxyphenyl)-2,3-dihydrochromen-4-one |
| 15,07_581,1807m/z | CCMSLIB00000845262 | C27H32O14 | 36,8 | -9,99471E+14 | NCGC00169797-03![3,4,5-trihydroxy-6-[4-hydroxy-2-[(1,2,3,6-tetrahydroxycyclohexanecarbonyl)oxymethyl]phenoxy]oxan-2-yl]methyl benzoate |
| 15,07_581,1807m/z | CCMSLIB00000845634 | C27H32O14 | 36,8 | -9,99471E+14 | NCGC00380866-01!(2S)-5,7-dihydroxy-2-(4-hydroxyphenyl)-6-[(2S,3R,4R,5S,6R)-3,4,5-trihydroxy-6-[[(2R,3R,4R,5R,6S)-3,4,5-trihydroxy-6-methyloxan-2-yl]oxymethyl]oxan-2-yl]-2,3-dihydrochromen-4-one |
| 15,07_581,1807m/z | CCMSLIB00000846433 | C27H32O14 | 36,8 | -9,99471E+14 | NCGC00169359-04!5-hydroxy-2-(4-hydroxyphenyl)-7-[(2S,3R,4S,5S,6R)-3,4,5-trihydroxy-6-[[(2R,3R,4R,5R,6S)-3,4,5-trihydroxy-6-methyloxan-2-yl]oxymethyl]oxan-2-yl]oxy-2,3-dihydrochromen-4-one |
| 15,07_581,1807m/z | CCMSLIB00000853023 | C27H32O14 | 36,8 | -9,99471E+14 | NCGC00385466-01_C27H32O14_(1S,4aS,6S,7aS)-6-Hydroxy-1-({6-O-[(2E)-3-(4-hydroxy-3,5-dimethoxyphenyl)-2-propenoyl]-beta-D-glucopyranosyl}oxy)-7-methylene-1,4a,5,6,7,7a-hexahydrocyclopenta[c]pyran-4-carboxylic acid |
| 15,07_581,1807m/z | CCMSLIB00004681457 | C34H28O9 | 38,5 | 1,28416E+14 | Actephilol B |
| 15,07_581,1807m/z | VF-NPL-QTOF000910 | C27H32O14 | 36,8 | -9,99471E+14 | (2S)-5,7-dihydroxy-2-(4-hydroxyphenyl)-6-[(2S,3R,4R,5S,6R)-3,4,5-trihydroxy-6-[[(2R,3R,4R,5R,6S)-3,4,5-trihydroxy-6-methyloxan-2-yl]oxymethyl]oxan-2-yl]-2,3-dihydrochromen-4-one |
| 15,07_581,1807m/z | VF-NPL-QTOF004936 | C27H32O14 | 36,9 | -9,99471E+14 | 7-[4,5-dihydroxy-6-(hydroxymethyl)-3-[(2S,3R,4R,5R,6S)-3,4,5-trihydroxy-6-methyloxan-2-yl]oxyoxan-2-yl]oxy-5-hydroxy-2-(4-hydroxyphenyl)-2,3-dihydrochromen-4-one |
| 15,07_581,1807m/z | VF-NPL-QTOF006186 | C27H32O14 | 36,8 | -9,99471E+14 | [3,4,5-trihydroxy-6-[4-hydroxy-2-[(1,2,3,6-tetrahydroxycyclohexanecarbonyl)oxymethyl]phenoxy]oxan-2-yl]methyl benzoate |
| 15,07_581,1807m/z | VF-NPL-QTOF009074 | C27H32O14 | 36,8 | -9,99471E+14 | Naringin |
| 15,07_440,1677m/z | CCMSLIB00000079780 | C24H25NO7 | 37,3 | -6,12142E+14 | "MLS001158477-01!(2Z)-2-[(2,4-dimethoxyphenyl)methylidene]-6-[2-[(2S)-2-(hydroxymethyl)pyrrolidin-1-yl]-2-oxoethoxy]-1-benzofuran-3-one" |
| 15,07_440,1677m/z | CCMSLIB00000080032 | C23H25N3O4S | 36 | 8,73617E+14 | MLS001146633-01! |
| 15,07_440,1677m/z | CCMSLIB00000080339 | C24H25NO7 | 37,3 | -6,12142E+14 | MLS001140978-01!4-[[[2-(8-methoxy-6-oxobenzo[c]chromen-3-yl)oxyacetyl]amino]methyl]cyclohexane-1-carboxylic acid |
| 15,07_440,1677m/z | CCMSLIB00000478453 | C25H21N5O3 | 36,4 | -9,16676E+14 | Quinadoline B_130007 |
| 16,48_324,1809m/z | CCMSLIB00000850968 | C17H25NO5 | 38,6 | 1,14787E+14 | NCGC00381005-01_C17H25NO5_ |
| 16,57_405,1399m/z | CCMSLIB00000847130 | C17H24O11 | 36,1 | 1,8724E+14 | NCGC00180103-03!methyl 5-hydroxy-7-(hydroxymethyl)-1-[3,4,5-trihydroxy-6-(hydroxymethyl)oxan-2-yl]oxy-1,4a,5,7a-tetrahydrocyclopenta[c]pyran-4-carboxylate |
| 16,57_405,1399m/z | CCMSLIB00000849857 | C17H24O11 | 36,1 | 1,8724E+14 | NCGC00169180-03_C17H24O11_Cyclopenta[c]pyran-4,7-dicarboxylic acid, 1-(beta-D-glucopyranosyloxy)-1,4a,5,6,7,7a-hexahydro-, 4-methyl ester, (1S,4aS,7S,7aS)- |
| 16,57_405,1399m/z | CCMSLIB00000852886 | C17H24O11 | 36,1 | 1,8724E+14 | NCGC00385275-01_C17H24O11_[2-(beta-D-Glucopyranosyloxy)-5-(methoxycarbonyl)-3-vinyl-3,4-dihydro-2H-pyran-4-yl]acetic acid |
| 16,57_405,1399m/z | CCMSLIB00000856145 | C17H24O11 | 36,1 | 1,8724E+14 | NCGC00169504-02_C17H24O11_Methyl 1-(hexopyranosyloxy)-7-hydroxy-7-(hydroxymethyl)-1,4a,7,7a-tetrahydrocyclopenta[c]pyran-4-carboxylate |
| 16,57_405,1399m/z | VF-NPL-QTOF002921 | C17H24O11 | 36,1 | 1,8724E+14 | 2-[3-ethenyl-5-methoxycarbonyl-2-[(2S,3R,4S,5S,6R)-3,4,5-trihydroxy-6-(hydroxymethyl)oxan-2-yl]oxy-3,4-dihydro-2H-pyran-4-yl]acetic acid |
| 16,57_405,1399m/z | VF-NPL-QTOF008483 | C17H24O11 | 36,1 | 1,8724E+14 | methyl (1S,4aR,7aR)-4a-hydroxy-7-(hydroxymethyl)-1-[(2S,3R,4S,5S,6R)-3,4,5-trihydroxy-6-(hydroxymethyl)oxan-2-yl]oxy-5,7a-dihydro-1H-cyclopenta[c]pyran-4-carboxylate |
| 16,57_412,1715m/z | CCMSLIB00000080284 | C23H25NO6 | 36,8 | -9,66615E+14 | "MLS001159032-01!2-[(4-ethyl-8,8-dimethyl-2-oxo-9,10-dihydropyrano[2,3-h]chromen-5-yl)oxy]-N-(furan-2-ylmethyl)acetamide" |
| 16,57_412,1715m/z | CCMSLIB00000080359 | C23H25NO6 | 36,8 | -9,66615E+14 | "MLS001141225-01!N-(furan-2-ylmethyl)-2-[(3,4,8,8-tetramethyl-2-oxo-9,10-dihydropyrano[2,3-h]chromen-5-yl)oxy]acetamide" |
| 17,10_306,1691m/z | CCMSLIB00000079032 | C17H23NO4 | 38 | -2,82849E+14 | CETRAXATE HCl |
| 17,41_333,0619m/z | VF-NPL-QTOF002647 | C16H12O8 | 37,6 | 4,34052E+14 | 2-(2,6-dihydroxy-4-methoxycarbonylbenzoyl)-3-hydroxybenzoic acid |
| 17,41_334,1368m/z | CCMSLIB00000852041 | C16H19N3O5 | 34,5 | -8,8713E+14 | NCGC00380446-01_C16H19N3O5_11-Hydroxy-11-isopropyl-4-methoxy-8-methyl-10,11-dihydro-6H-oxepino[2,3-d]pyrazino[1,2-a]pyrimidine-6,9(8H)-dione |
| 17,58_497,2597m/z | CCMSLIB00000846747 | C22H40O12 | 35 | 9,71903E+14 | NCGC00380959-01![(2R,3S,4S,5R,6S)-3,4,5-trihydroxy-6-[(2S,3R,4S,5S,6R)-3,4,5-trihydroxy-6-(hydroxymethyl)oxan-2-yl]oxyoxan-2-yl]methyl 8-methylnonanoate |
| 17,58_585,1835m/z | CCMSLIB00000845639 | C26H32O15 | 35,5 | 3,62244E+14 | NCGC00180492-02![2-[(2S,3R,4S,5S,6R)-3,4,5-trihydroxy-6-(hydroxymethyl)oxan-2-yl]oxyphenyl]methyl 2-hydroxy-6-[(2S,3R,4S,5S,6R)-3,4,5-trihydroxy-6-(hydroxymethyl)oxan-2-yl]oxybenzoate |
| 17,58_585,1835m/z | CCMSLIB00000854335 | C26H32O15 | 35,5 | 3,62244E+14 | NCGC00386039-01_C26H32O15_Cyclopenta[c]pyran-4-carboxylic acid, 7-[[[(2E)-3-(3,4-dihydroxyphenyl)-1-oxo-2-propen-1-yl]oxy]methyl]-1-(hexopyranosyloxy)-1,4a,5,6,7,7a-hexahydro-5,6-dihydroxy-, methyl ester |
| 17,58_481,2832m/z | CCMSLIB00000851545 | C26H40O8 | 36,2 | 7,46211E+14 | NCGC00169477-02_C26H40O8_2(5H)-Furanone, 3-[2-[(1R,4aS,5R,8aS)-5-[(beta-D-glucopyranosyloxy)methyl]decahydro-5,8a-dimethyl-2-methylene-1-naphthalenyl]ethyl]- |
| 17,58_481,2832m/z | VF-NPL-QTOF009088 | C26H40O8 | 36,2 | 7,46211E+14 | neoandrographolide |
| 17,86_582,1651m/z | CCMSLIB00000079961 | C32H26ClN4O5 | 33,3 | -2,26953E+14 | MLS001140668-01! |
| 17,86_495,2636m/z | CCMSLIB00000080176 | C27H34N4O5 | 33 | 6,86036E+14 | "MLS001158206-01!(2S)-2-[[(2S)-4-methyl-2-[[(2S)-3-oxo-2-propan-2-yl-2,4-dihydroquinoxaline-1-carbonyl]amino]pentanoyl]amino]-3-phenylpropanoic acid" |
| 17,98_400,2150m/z | CCMSLIB00000079598 | C23H29NO5 | 37,3 | 8,01172E+14 | "MLS001142750-01!7-[1-[(4aS,8aS)-4a-hydroxy-1,3,4,5,6,7,8,8a-octahydroisoquinolin-2-yl]-1-oxopropan-2-yl]oxy-4,8-dimethylchromen-2-one" |
| 18,14_348,1741m/z | CCMSLIB00000078109 | C18H24ClN4O | 32,6 | 8,60994E+14 | Granisetron HCl |
| 18,14_348,1741m/z | CCMSLIB00000085243 | C18H24ClN4O | 32,6 | 8,60994E+14 | MLS001401373-01!Granisetron??Hydrochloride |
| 19,21_454,1841m/z | CCMSLIB00000085566 | C20H27N3O9-2 | 37,9 | 4,53768E+14 | MLS002154064-01!Xamoterol hemifumarate73210-73-8 |
| 19,36_414,1035m/z | CCMSLIB00000080144 | C21H20ClN3O2S | 33,9 | -5,77463E+14 | MLS001146623-01! |
| 19,36_430,2626m/z | CCMSLIB00000851037 | C25H35NO5 | 37,3 | 8,80767E+14 | NCGC00381123-01_C25H35NO5_Spiro[2H-furo[2,3-e]isoindole-2,1'(2'H)-naphthalen]-6(3H)-one, 3',4',4'a,5',6',7,7',8,8',8'a-decahydro-4,6'-dihydroxy-7-(2-hydroxyethyl)-2',5',5',8'a-tetramethyl-, (2R,2'R,6'R,8a'S)- |
| 19,36_430,2626m/z | CCMSLIB00005436406 | C25H35NO5 | 37,3 | 8,80767E+14 | (2R,2'R,4a'S,6'R,8a'S)-4,6'-dihydroxy-7-(2-hydroxyethyl)-2',5',5',8a'-tetramethyl-3',4',4a',5',6',7,7',8,8',8a'-decahydro-2'H-spiro[furo[2,3-e]isoindole-2,1'-naphthalen]-6(3H)-one |
| 19,36_430,2626m/z | VF-NPL-QTOF001568 | C25H35NO5 | 37,3 | 8,80767E+14 | (3R,7R,8R,8aS)-3,4'-dihydroxy-7'-(2-hydroxyethyl)-4,4,7,8a-tetramethylspiro[2,3,4a,5,6,7-hexahydro-1H-naphthalene-8,2'-3,8-dihydrofuro[2,3-e]isoindole]-6'-one |
| 19,54_584,3382m/z | CCMSLIB00000478066 | C37H45NO5 | 32,7 | 1,98124E+14 | Shearinine F_120146 |
| 19,54_584,3382m/z | CCMSLIB00000478067 | C37H45NO5 | 32,7 | 1,98124E+14 | Shearinine A_120147 |
| 19,54_539,2909m/z | CCMSLIB00000085272 | C29H38N4O6 | 32,3 | 8,27292E+14 | MLS001334042-01!WAY-100635 maleate salt |
| 19,67_329,2693m/z | CCMSLIB00000845026 | C19H36O4 | 35,6 | 1,88198E+14 | NCGC00385642-01!1,4-dihydroxyheptadec-16-en-2-yl acetate |
| 19,67_329,2693m/z | VF-NPL-QTOF002611 | C19H36O4 | 35,6 | 1,88198E+14 | 2,4-dihydroxyheptadec-16-enyl acetate |
| 20,11_348,1526m/z | CCMSLIB00000001600 | C17H21N3O5 | 36,9 | -7,93563E+14 | Oxepinamide A |
| 20,68_308,1852m/z | CCMSLIB00000078700 | C17H25NO4 | 39,2 | -1,37156E+14 | NCGC00160253-01!1-(2-hydroxy-3-methyl)-butyl-hydrocotarnine |
| 20,68_308,1852m/z | CCMSLIB00000079034 | C17H25NO4 | 39,2 | -1,37156E+14 | Buflomedil HCl |
| 21,32_426,1878m/z | CCMSLIB00000079371 | C24H27NO6 | 37 | -7,85335E+14 | "MLS001158260-01!N-[2-(3,4-dimethoxyphenyl)ethyl]-2-(3,4,7-trimethyl-2-oxochromen-5-yl)oxyacetamide" |
| 24,79_323,2220m/z | CCMSLIB00000845736 | C19H30O4 | 39 | 1,07732E+12 | NCGC00380425-01!1-(3,5-dihydroxyphenyl)-12-hydroxytridecan-2-one |
| 24,79_323,2220m/z | VF-NPL-QTOF007133 | C19H30O4 | 39 | 1,07732E+14 | Clovanediol Diacetate |
| 34,23_537,3016m/z | CCMSLIB00000853372 | C29H44O9 | 32,5 | -7,91415E+14 | NCGC00384523-01_C29H44O9_Card-20(22)-enolide, 3-(beta-D-glucopyranosyloxy)-14-hydroxy-, (3beta,9xi)- |
| 36,66_524,3205m/z | CCMSLIB00005465257 | C28H45NO8 | 33,8 | -2,50996E+14 | "((4R)-4-((3R,5S,7R,9S,10S,12S,13R,14S,17R)-3,7,12-trihydroxy-10,13-dimethylhexadecahydro-1H-cyclopenta[a]phenanthren-17-yl)pentanoyl)-L-aspartic acid" |
| 36,66_524,3205m/z | CCMSLIB00005465874 | C28H45NO8 | 33,8 | -2,50996E+14 | Aspartate conjugated cholic acid |
| 37,51_568,3198m/z | CCMSLIB00000078950 | C32H45N3O4S | 32,9 | -9,52063E+14 | NELFINAVIR MESYLATE |
| 37,51_568,3198m/z | CCMSLIB00000085934 | C32H45N3O4S | 32,9 | -9,52063E+14 | MLS001195634-01!nelfinavir |
| 37,51_568,3198m/z | CCMSLIB00004679928 | C38H39N4O+ | 32,9 | 2,66041E+14 | 3',4',5',6'-tetradehydrolongicaudatine Y |
| 38,82_378,2220m/z | CCMSLIB00005489305 | C14H29N6O6+ | 36,4 | -4,62234E+13 | hexamethylolmelamine pentamethyl ether (putative) |
| 45,85_409,1807m/z | CCMSLIB00000079063 | C22H29ClO5 | 33 | 7,4109E+14 | Beclomethasone |
| 45,85_409,1807m/z | CCMSLIB00000086182 | C22H29ClO5 | 33 | 7,4109E+14 | MLS001076089-01! |
